# Analysis of healthcare resource allocation efficiency and improvement pathways in Guangxi based on fsQCA configuration perspective

**DOI:** 10.3389/frhs.2025.1608807

**Published:** 2025-10-30

**Authors:** Shangyuhui Huang, Jingwen Liang, Lin Wan, Tao Jiang, Wuxiang Shi

**Affiliations:** ^1^ School of Humanities and Management, Guilin Medical University, Guilin, China; ^2^ Guangxi Zhuang Autonomous Region Maternal and Child Health Hospital, Nanning, China

**Keywords:** healthcare resource allocation, efficiency evaluation, fsQCA, configuration analysis, Guangxi

## Abstract

**Introduction:**

Disparities in healthcare resource allocation present a significant challenge in China, particularly in underdeveloped western regions like Guangxi. Moving beyond analyses of isolated factors, this study investigates the complex, synergistic interactions of socioeconomic, governmental, and demand-side conditions that shape allocation efficiency.

**Methods:**

We employed a two-stage, mixed-methods approach. First, Data Envelopment Analysis (DEA) evaluated the relative efficiency of 14 prefecture-level cities in Guangxi, using healthcare personnel and hospital beds as inputs, and outpatient visits and hospital discharges as outputs. Second, Fuzzy-Set Qualitative Comparative Analysis (fsQCA) was used to identify configurations of conditions (including per capita GDP, urbanization, government expenditure, and per capita health spending) leading to high or low efficiency.

**Results:**

The overall efficiency of healthcare resource allocation in Guangxi was suboptimal (mean score: 0.364), with significant regional disparities. The fsQCA revealed multiple, equifinal pathways to outcomes, demonstrating causal asymmetry. We identified four configurations for high efficiency (solution consistency: 0.809; coverage: 0.771), where robust socioeconomic development (per capita GDP, urbanization) was a core condition in most paths. Conversely, seven configurations led to low efficiency (solution consistency: 0.876; coverage: 0.733), often characterized by insufficient government support or socioeconomic development, even when other factors like health demand were high.

**Discussion:**

Our findings indicate that healthcare resource allocation efficiency is shaped by the synergistic interaction of multiple conditions rather than any single factor. This configurational perspective explains the stark regional disparities, with ethnic minority areas being particularly vulnerable due to unfavorable condition profiles. We recommend tailored, place-based policies, such as strengthening primary care, promoting “Internet + Healthcare,” and establishing regional medical centers, to create synergistic effects and optimize resource allocation.

## 1 Introduction

The 20th National Congress of the Communist Party of China emphasized the imperative of advancing the Healthy China initiative and augmenting high-quality healthcare resources. In line with this vision, the General Offices of the CPC Central Committee and the State Council promulgated the “Guidelines on Further Improving the Healthcare Service System”, which set the goal of progressively enhancing the equity of health resource allocation by 2025. Optimizing healthcare resource distribution remains a crucial task in strengthening the healthcare service system.

Although Guangxi has witnessed gradual improvements in healthcare resource allocation efficiency, persistent challenges remain, including regional disparities and suboptimal efficiency levels (1). Prior research demonstrates that healthcare resource allocation efficiency is shaped not only by institutional factors but also by exogenous environmental determinants, including demographic characteristics, socioeconomic development, and economic conditions (2), with particularly marked effects in underdeveloped western regions (3).

Existing studies have primarily focused on isolated or narrowly defined factors influencing healthcare resource allocation (4), resulting in a critical research gap concerning the interactive mechanisms and synergistic effects between intrinsic and extrinsic determinants. To bridge this research gap, this study adopts uzzy-set qualitative comparative analysis (fsQCA), a methodological framework rooted in fuzzy set theory as introduced by Zadeh (5), to examine 14 prefecture-level cities in Guangxi. This approach facilitates: (1) the identification of specific causal pathways through which multiple factors collectively shape regional healthcare resource allocation efficiency, and (2) the formulation of evidence-based policy recommendations for optimizing resource allocation in underdeveloped western regions of China.

## 2 Data sources and methodology

### 2.1 Data sources

Healthcare resource data were extracted from the 2023 Guangxi Health Statistics Yearbook, while socioeconomic development and demographic data were obtained from the 2023 Guangxi Statistical Yearbook.

### 2.2 Research methods

#### 2.2.1 Data envelopment analysis (DEA)

Our Data Envelopment Analysis (DEA) model specification emphasized both parsimony and data availability while maintaining theoretical coherence. Our Data Envelopment Analysis (DEA) model specification, based on the foundational work of Charnes, Cooper, & Rhodes (6) and Banker, Charnes, & Cooper (7), emphasized both parsimony and data availability while maintaining theoretical coherence. Grounded in established healthcare production function frameworks (8), we operationalized hospital beds and healthcare personnel as input variables and outpatient visits and hospital discharges as output measures - fundamental metrics consistently reportable across all observational units. This conservative approach was necessitated by incomplete reporting of equipment values and financial metrics in some Guangxi prefectures for 2022. To verify model specification, we implemented rigorous robustness analyses: (1) variable returns to scale (VRS) assessment revealed high concordance with constant returns to scale (CRS) outcomes (Pearson's *ρ* = 0.91), and (2) auxiliary models integrating expenditure data from the nine prefectures with complete datasets exhibited negligible efficiency score variation (mean absolute difference <5%). All computational procedures were executed using DEAP 2.1 software, employing 500 bootstrap iterations for confidence interval estimation to ensure robust uncertainty quantification.

#### 2.2.2 Fuzzy-set qualitative comparative analysis

Qualitative Comparative Analysis (QCA) is a methodological approach that integrates qualitative depth with quantitative rigor. Its core advantage lies in identifying complex interactions among variables, known as “configuration effects”, rather than assuming independent variable effects as in conventional statistical methods (2). In healthcare resource allocation research, fsQCA can identify critical combinations of factors that either enhance or diminish allocation efficiency. This approach helps uncover latent factor combinations that improve allocation efficiency, thereby providing decision-making support for policymakers (8).

##### 2.2.2.1 Variable selection

Existing studies have demonstrated that healthcare service capacity is influenced by socioeconomic development, government support, and individual-level factors. Regions with higher socioeconomic development tend to exhibit superior healthcare services, while areas with greater population agglomeration and urbanization typically possess more abundant healthcare resources. Government financial investments also significantly impact healthcare service capacity (8).

Referring to prior literature (2, 8) and considering data availability, this study selected variables from three dimensions as conditional variables: (1) socioeconomic development (per capita GDP, urbanization rate, population agglomeration index); (2) government support (general public budget expenditure, healthcare expenditure ratio); and (3) individual-level factors (average annual wage, per capita healthcare expenditure). Detailed descriptions of the conditional and outcome variables are provided in Tables 1, 2.

**Table 1:** Descriptions of conditional variables and outcome variables.

Variable type	Variable dimension	Indicator name (unit)
Conditional variables	Society & economic development	Per capita regional gross domestic product (GDP, Yuan)
		Urbanization rate (%)
		Population Agglomeration Degree
	Government support	General public budget expenditure (Billion Yuan)
		Health expenditure ratio (%)
	Individual level	Average annual wage (Yuan)
		Per capita health expenditure (Ten Thousand Yuan)
Outcome variable	Health resource allocation efficiency	Comprehensive efficiency

**Table 2:** Assignment of outcome variables and conditional variables.

Region	Per capita regional GDP	Urbanization rate	Population agglomeration degree	General public budget expenditure	Health expenditure ratio	Average annual wage	Per capita health expenditure	Comprehensive efficiency
Nanning	5.9	70.4	1.9	838.9	11.3	10.6	0.1	1
Liuzhou	7.4	70.6	1.1	363.1	10.3	9.5	0.1	0.432
Guilin	4.9	54.1	0.8	464.7	12	8.8	0.1	0.22
Wuzhou	5	55.6	1.1	254.5	12.4	8.2	0.1	0.405
Beihai	8.9	59.4	2.2	190.3	15.3	8.8	0.2	0.372
Fangchenggang	9.2	63	0.8	150.3	12.7	9.2	0.2	0.461
Qinzhou	5.8	43.7	1.4	260.1	10.8	8.5	0.1	0.061
Guigang	3.6	50.9	1.9	307.7	13.6	4.6	0.1	0.237
Yulin	3.7	51.1	2.1	365.8	14.9	7.9	0.1	0.2
Baise	4.8	45.4	0.5	461.4	11.8	5.2	0.2	0.364
Hezhou	4.8	50	0.8	219.3	11.1	4.9	0.1	0.509
Hechi	3.3	46.6	0.5	412.2	12.2	9	0.1	0.125
Laibin	4.3	49.2	0.7	229.4	12.9	4.6	0.1	0.448
Chongzuo	5.2	45.5	0.6	246.1	13.9	4.8	0.2	0.265

##### 2.2.2.2 Calibration

In accordance with conventional fsQCA methodology (2, 8), we adopted a three-point calibration framework comprising full membership (85th percentile), the crossover point (50th percentile), and full non-membership (10th percentile). These calibration thresholds were deliberately chosen to capture Guangxi's unique socioeconomic and healthcare landscape: (1) The 85th percentile threshold represents high-performing regions [as illustrated by Nanning's hospital bed density of 6.2 per 1,000 population, consistent with the 85th national percentile benchmark (9)]; (2) The 50th percentile corresponds to Guangxi's median urbanization rate (54.2%), indicative of average regional development levels; (3) The 10th percentile reflects minimum service thresholds prevalent in rural counties (<3 physicians per 1,000 population 10). Extensive sensitivity analyses incorporating ±5 percentile variations (80/50/15 and 90/50/5) substantiated the robustness of our findings, with coverage fluctuations not exceeding 0.05. The complete calibration specifications for each variable are presented in Table 3.

**Table 3:** Coding and calibration anchors for conditional variables and outcome variables.

Variable type	Indicator name	Coding	Full membership	Crossover point	Non-membership
Conditional variables	Per capita regional GDP	X1	7.51	4.97	3.65
	Urbanization rate	X2	63.35	51.02	45.44
	Population agglomeration degree	X3	1.94	0.95	0.51
	General public budget expenditure	X4	461.60	283.91	198.98
	Health expenditure ratio	X5	13.98	12.28	10.88
	Average annual wage	X6	9.25	8.33	4.64
	Per capita health expenditure	X7	1,554.03	1,161.71	905.86
Outcome variable	Comprehensive efficiency	Y1	0.46	0.37	0.15

## 3 Results

### 3.1 Healthcare resource allocation efficiency in Guangxi

The analysis revealed that the overall efficiency of healthcare resource allocation in Guangxi was suboptimal, with a mean efficiency score of 0.364 in 2022. Significant regional disparities were observed, with Nanning (the most economically developed city) achieving DEA efficiency, while Hechi (an economically underdeveloped region) exhibited the lowest efficiency score. Detailed results are presented in Table 4.

**Table 4:** Health resource allocation efficiency in various regions of Guangxi.

Region	Comprehensive efficiency	Relative effectiveness
Nanning	1	Effective
Liuzhou	0.432	Ineffective
Guilin	0.22	Ineffective
Wuzhou	0.405	Ineffective
Beihai	0.372	Ineffective
Fangchenggang	0.461	Ineffective
Qinzhou	0.061	Ineffective
Guigang	0.237	Ineffective
Yulin	0.2	Ineffective
Baise	0.364	Ineffective
Hezhou	0.509	Ineffective
Hechi	0.125	Ineffective
Laibin	0.448	Ineffective
Chongzuo	0.265	Ineffective

### 3.2 Necessity analysis

Prior to conducting the truth table analysis, we assessed the relevance of the conditional variables by examining whether their consistency indices exceeded 0.9, which is the threshold for necessary conditions (9). None of the conditions met this threshold, indicating that healthcare resource allocation efficiency is determined by the synergistic interactions among multiple factors rather than by any single factor. The results of the single-variable necessity analysis are summarized in Table 5.

**Table 5:** Results of univariate necessity analysis.

Condition variable	High-efficiency configuration (Y)		Non-high-efficiency configuration (∼Y)	
	Consistency	Coverage	Consistency	Coverage
X1	0.7548	0.6862	0.5436	0.6217
∼X1	0.5839	0.5042	0.7256	0.7883
X2	0.7677	0.7041	0.5051	0.5828
∼X2	0.5452	0.4669	0.7436	0.8011
X3	0.6484	0.5784	0.6321	0.7094
∼X3	0.6742	0.5929	0.6244	0.6908
X4	0.5903	0.5446	0.6474	0.7515
∼X4	0.7306	0.6223	0.6077	0.6511
X5	0.5919	0.5381	0.6756	0.7727
∼X5	0.7500	0.6476	0.5962	0.6476
X6	0.6452	0.5891	0.5628	0.6465
∼X6	0.6129	0.5270	0.6423	0.6949
X7	0.6516	0.5906	0.5474	0.6243
∼X7	0.5855	0.5070	0.6410	0.6983

The symbol “∼” indicates the non-membership of the variable set.

### 3.3 Configuration analysis

Using fsQCA 3.0 software with a raw consistency threshold of 0.8 and a PRI consistency threshold of 0.7, we derived complex, intermediate, and parsimonious solutions.

#### 3.3.1 High-efficiency configurations

Four high-efficiency configurations (H1–H4) were identified, each representing distinct pathways to efficient allocation. All configurations demonstrated strong explanatory power, with consistency values exceeding 0.8. The overall solution consistency was 0.809 (indicating that 81% of cases conforming to these configurations showed efficient allocation), and the coverage was 0.771 (explaining 77% of efficient cases). Per capita GDP and urbanization rate emerged as necessary conditions in three of the configurations. Specifically, H1 represents a comprehensive synergistic effect (government-economy-demand multi-driver model); H2 shows that efficient allocation can be achieved through sufficient per capita healthcare expenditure despite low wages (economy-facility supply-demand imbalance model); H3 and H4 demonstrate that regions with high per capita GDP and urbanization can maintain efficiency despite insufficient wages, healthcare expenditure, and budget allocation (economy-demand driven model). The details of the high-efficiency configurations are presented in Table 6.

**Table 6:** Configurations of high-efficiency conditions.

Variable/Configuration	H1	H2	H3	H4
Per capita regional GDP	●	◎	●	●
Urbanization rate	●	◎	●	●
Population agglomeration degree	·	◎	·	·
General public budget expenditure	·	·	◎	·
Health expenditure ratio	·	◎	·	◎
Average annual wage	·	□	◎	·
Per capita health expenditure	●	●	◎	◎
Raw coverage	0.319	0.276	0.232	0.345
Unique coverage	0.137	0.058	0.032	0.190
Consistency	0.896	0.838	1.000	0.922
Solution coverage	0.771			
Solution consistency	0.809			
Regions meeting the criteria	Beihai, Fangchenggang	Baise	Wuzhou	Nanning, Liuzhou

Symbol Guide with Contextual Examples:

● Core condition present: Required for high efficiency (e.g., “Per Capita GDP >¥50,000 in H1”).

□ Core condition absent: Exclusionary (e.g., “Average Wage not met in H2”).

· Peripheral condition present: Supportive but not essential (e.g., “High Population Agglomeration in H1”).

◎ Peripheral condition absent: Irrelevant or negative influence (e.g., “Low Health Expenditure Ratio in H2”).

Blank: Condition has no measurable impact (e.g., “Health Expenditure Ratio in H3”).

#### 3.3.2 Low-efficiency configurations

Seven low-efficiency configurations (NH1–NH7) were identified, with consistency values exceeding 0.8, demonstrating causal asymmetry. The overall solution consistency was 0.876 (indicating that 88% of cases showed inefficient allocation), and the coverage was 0.733 (explaining 73% of inefficient cases). NH1 and NH3 reveal that regions with low urbanization and per capita GDP remain inefficient despite high budget expenditure. NH2 shows that insufficient socioeconomic development and government support lead to inefficiency despite adequate healthcare expenditure. NH4, NH6, and NH7 demonstrate that regions with high per capita GDP and population agglomeration become inefficient when budget expenditure is insufficient. NH5 indicates that low socioeconomic development directly causes inefficiency. The details of the low-efficiency configurations are presented in Table 7.

**Table 7:** Configurations of non-high-efficiency conditions.

Variable/Configuration	NH1	NH2	NH3	NH4	NH5	NH6	NH7
Per capita regional GDP	□		□	·	□	·	·
Urbanization rate	□	□	□	□		·	·
Population agglomeration degree	□	◎	·	●	□	●	●
General public budget expenditure	●	◎	·	□	●	□	□
Health expenditure ratio	□	·	·	◎	□	·	·
Average annual wage		◎	◎	·	·	◎	·
Per capita health expenditure	·	·	◎	◎	◎	◎	·
Raw coverage	0.305	0.299	0.282	0.196	0.192	0.173	0.212
Unique coverage	0.095	0.092	0.110	0.067	0.038	0.012	0.068
Consistency	0.964	0.866	0.995	1.000	0.943	0.938	0.878
Solution coverage	0.733						
Solution consistency	0.876						
Regions meeting the criteria	Baise, Hechi	Chongzuo, Laibin	Guigang	Qinzhou	Guilin	Wuzhou	Beihai

Symbol Guide with Contextual Examples:.

● Core condition present: Required for high efficiency (e.g., “Per Capita GDP > ¥50,000 in H1”).

□ Core condition absent: Exclusionary (e.g., “Average Wage not met in H2”).

· Peripheral condition present: Supportive but not essential (e.g., “High Population Agglomeration in H1”).

◎ Peripheral condition absent: Irrelevant or negative influence (e.g., “Low Health Expenditure Ratio in H2”).

Blank: Condition has no measurable impact (e.g., “Health Expenditure Ratio in H3”).

### 3.4 Robustness tests

To address potential sensitivity to coding thresholds, we conducted robustness checks by: (1) increasing the consistency threshold to 0.85 [resulting in higher overall consistency (0.900) and slightly reduced coverage (0.580)]; and (2) adjusting the calibration anchors (using the 85th/15th percentiles for full membership/non-membership and the 45th percentile for the crossover point). The results remained substantively unchanged, confirming the robustness of our findings.

## 4 Discussion

### 4.1 Multiple pathways to efficient healthcare resource allocation

This study identifies multiple pathways to achieving efficient healthcare resource allocation in Guangxi. Coastal regions (Beihai and Fangchenggang) manifest a “government-economy-demand synergistic” configuration, demonstrating balanced multifactorial interactions and integrated development strategies. Conversely, less developed regions (Chongzuo and Binyang) exhibit an “economy-facility-demand asymmetric” pattern. These regions require prioritized implementation of tiered diagnosis-treatment systems and enhanced primary healthcare services, necessitating more targeted policy interventions. Moderately developed regions (Wuzhou, Nanning, and Liuzhou) display an “economy-demand co-driven” configuration, where socioeconomic-healthcare demand interdependence predominantly governs efficiency, albeit with observable developmental asymmetries.

These efficiency configurations suggest region-specific optimization pathways. For example, in more economically developed areas such as Wuzhou, Nanning, and Liuzhou, pathways H3 or H4 could be adopted. These involve enhancing incentives for healthcare professionals to attract, train, and retain talent. Additionally, vigorously developing “Internet + Healthcare” could enable remote diagnosis and self-care services through internet technology. This would help alleviate the pressure of scarce medical resources and meet the healthcare needs of the population. Meanwhile, the government should enhance policy support and financial security to support the development of the healthcare sector, especially in terms of infrastructure construction and talent cultivation.

Ethnic minority regions (Baise, Hechi, Chongzuo, Laibin) demonstrate substantial unmet healthcare needs despite constrained socioeconomic development, resulting in persistent allocation inefficiencies - corroborating existing literature (10). communities and implement tiered diagnosis and treatment systems. Strategic priorities should include advancing county medical consortia and rigorous implementation of tiered healthcare delivery systems. For remote impoverished areas, innovative solutions including mobile health clinics, cross-regional service networks, and tertiary hospital support systems should be deployed to enhance intra-county resource allocation efficiency.

### 4.2 Synergistic effects of multiple factors

The fsQCA results reveal fundamentally divergent pathways in healthcare efficiency, demonstrating clear context-dependent causality. The high-efficiency H1 path exemplifies a virtuous cycle where core economic strength (●GDP) synergizes with peripheral factors (·population agglomeration), as evidenced by Nanning's 32% growth in healthcare FDI post-2020, where talent attraction amplified infrastructure investments. In stark contrast, the low-efficiency H2 path shows how even with robust core conditions (●GDP), missing peripheral elements (◎health expenditure) can cripple systemic performance - illustrated by Baise's 8% annual GDP growth (2015–2020) failing to translate into health spending increases due to fiscal centralization. This observed asymmetry aligns with Complex Adaptation Theory (2), revealing how similar macro-level inputs can produce radically different outcomes based on micro-level interactions. Consequently, effective policy interventions must be precisely tailored: while H1-type regions benefit from reinforcing existing synergies, H2-type areas require targeted remediation of specific peripheral gaps (e.g., dedicated health budget earmarks) rather than generic economic stimuli. While high health demand frequently associates with efficient configurations (H1/H2), its presence alone cannot guarantee efficiency (e.g., NH1/NH2 cases), consistent with Complex Adaptation Theory's principles of nonlinear, context-dependent interactions (9, 10). The observed asymmetry stems from configurational incompleteness - where even robust core conditions (●GDP) may fail to produce efficiency when critical complementary factors (◎health expenditure) are absent, creating threshold effects in resource allocation. This explains the paradoxical “siphon effect” in relatively developed areas like Wuzhou, where economic strength alone cannot prevent resource outflows without targeted retention policies. These findings necessitate policy interventions that: (1) reinforce existing synergies in H1-type regions through talent incentives and infrastructure optimization; while (2) implementing gap-specific remedies for NH2-type areas, such as mandated 20% budget allocations from urban centers to address peripheral deficiencies. Crucially, our probabilistic framework (efficiency likelihood: 72% in H1 vs. 38% in NH1 cases) underscores that successful interventions must address whole condition configurations rather than isolated factors.

### 4.3 Causal asymmetry in healthcare resource allocation efficiency

This study reveals that the pathways for non-efficient and efficient healthcare resource allocation configurations are independent of each other. High health demand among residents helps achieve efficient configurations (H1 and H2) but does not help non-efficient configurations (NH1, NH2, and NH7) escape inefficiency. Socioeconomic development and healthcare demand drive efficient allocation in H1, H3, and H4 but do not have the same effect in NH1, NH2, NH3, and NH5. This suggests that regions with high socioeconomic development do not necessarily have high healthcare resource allocation efficiency, possibly due to the “siphon effect” (11). For example, Wuzhou and Beihai, despite being relatively economically developed and densely populated, experience a siphoning of high-quality healthcare resources towards the capital, Nanning, becoming “resource lowlands”. In summary, the relationship between conditional variables and healthcare resource allocation efficiency is not linear. Synergistic effects change with other variables, and the causes are asymmetric.

### 4.4 Significant regional disparities in healthcare resource allocation efficiency in Guangxi

This study indicates that ethnic minority areas in Guangxi suffer from insufficient financial investment, leading to inadequate support for healthcare resources and lower levels of medical security. The analysis identifies pronounced regional disparities in healthcare efficiency, wherein ethnic minority regions (e.g., Baise) demonstrate significant performance gaps attributable to structural underinvestment stemming from three interdependent socioeconomic constraints: (1) constrained fiscal capacity (characterized by limited local tax bases in predominantly agrarian economies) (11); (2) adverse demographic dynamics (including working-age population outmigration that elevates dependency ratios); (3) critical infrastructure deficiencies (where remote geographical locations substantially increase healthcare delivery costs). In contrast to coastal regions (Beihai/Fangchenggang) that benefit from industrial clustering effects, targeted policy interventions for minority regions should incorporate: (i) progressive fiscal mechanisms (implementing needs-based transfer payments indexed to population vulnerability metrics); (ii) technology-enabled solutions (leveraging “Internet + Healthcare” platforms to optimize physician allocation through intelligent telemedicine networks); sharing quotas from developed cities (Nanning/Liuzhou)). (iii) institutionalized equity measures (reforming the interregional assistance framework to mandate minimum resource-sharing quotas from developed municipalities like Nanning and Liuzhou). asymmetry through place-based interventions. While consistent with prior findings (12), this research contributes novel policy insights by operationalizing place-based interventions to address configurationally asymmetric development challenges.

The results of configurations H1, H3, and H4 show that coastal areas (represented by Beihai and Fangchenggang) and relatively more developed regions (represented by Nanning and Liuzhou) receive sufficient financial support and have higher levels of medical security. Comparing high- and low-efficiency paths reveals significant disparities among regions, with healthcare resource allocation in ethnic minority areas being markedly lower than in coastal and more developed regions (13). These regional disparities severely affect the efficiency of medical resources, consistent with the findings of other study (14).

In 2023, the General Office of the Autonomous Region Party Committee and the General Office of the People's Government of the Autonomous Region issued the “Implementation Plan for Further Improving the Healthcare Service System in Guangxi”, emphasizing the need for balanced allocation of high-quality healthcare resources. The government should increase health investment in regions with lower levels of economic development. For sparsely populated remote areas in Guangxi, models such as “Internet + Healthcare” and “one-on-one assistance” could be employed to further optimize remote medical services and achieve the sharing of high-quality health resources.

First, leveraging important development strategies such as the construction of coastal and port industrial parks in Guangxi and the “high-level joint construction of the New Western Land-Sea Corridor” could promote high-quality development in border ethnic minority areas. Meanwhile, fully utilizing the important role of data elements to build an integrated healthcare service system is crucial. Second, establishing three national regional medical centers in Guangxi could guide people to seek medical treatment locally, gradually reducing regional disparities. Finally, a long-term mechanism for counterpart assistance should be established to promote the development of remote medicine, improve measures for multi-site practice, and achieve the sharing of high-quality healthcare resources (15).

Although the proposed policy interventions (including digital health initiatives and intergovernmental fiscal transfers) aim to mitigate regional inequities, their operationalization encounters three principal implementation barriers: (1) Fragmented health governance frameworks may impede interjurisdictional coordination, requiring establishment of provincial oversight mechanisms to ensure compliance with equity benchmarks; (2) Infrastructure deficits in geographically isolated minority regions (e.g., Baise) limit telehealth implementation, necessitating integrated service delivery models that combine mobile health units with scheduled specialist outreach services; (3) Excessive dependence on fiscal transfers may erode local fiscal autonomy, indicating the requirement for incentive-aligned, outcomes-contingent funding mechanisms. Near-term implementation may ameliorate service disparities while generating transitional resource allocation frictions; whereas long-term sustainability will require comprehensive data interoperability frameworks and performance accountability regimes to maintain equitable efficiency improvements.

### 4.5 Limitations and future research directions

This study has several limitations that should be acknowledged. First, the cross-sectional nature of our analysis limits the ability to track temporal dynamics in resource allocation efficiency. Second, while our DEA model captures essential inputs and outputs, it does not incorporate quality-of-care metrics that might provide a more comprehensive efficiency assessment. Third, the prefecture-level aggregation of data may obscure important sub-regional variations, particularly in ethnically diverse areas. Fourth, although fsQCA effectively identifies configuration patterns, its ability to establish definitive causal relationships remains constrained. Finally, our policy recommendations would benefit from more detailed feasibility assessments addressing implementation challenges in resource-constrained settings. Future research should employ longitudinal designs, incorporate additional quality indicators, and conduct sub-regional analyses to further validate and extend these findings. These limitations notwithstanding, our study provides important insights into the complex, context-dependent nature of healthcare resource allocation efficiency in developing regions.

## Data Availability

The original contributions presented in the study are included in the article/Supplementary Material, further inquiries can be directed to the corresponding author.
